# Predicting financial trouble using call data—On social capital, phone logs, and financial trouble

**DOI:** 10.1371/journal.pone.0191863

**Published:** 2018-02-23

**Authors:** Rishav Raj Agarwal, Chia-Ching Lin, Kuan-Ta Chen, Vivek Kumar Singh

**Affiliations:** 1 Institute of Information Science, Academia Sinica, Taipei, Taiwan; 2 School of Communication and Information, Rutgers University, New Brunswick, New Jersey, United States of America; 3 Media Labs, Massachusetts Institute of Technology, Cambridge, Massachusetts, United States of America; University of Oxford, UNITED KINGDOM

## Abstract

An ability to understand and predict financial wellbeing for individuals is of interest to economists, policy designers, financial institutions, and the individuals themselves. According to the Nilson reports, there were more than 3 billion credit cards in use in 2013, accounting for purchases exceeding US$ 2.2 trillion, and according to the Federal Reserve report, 39% of American households were carrying credit card debt from month to month. Prior literature has connected individual financial wellbeing with social capital. However, as yet, there is limited empirical evidence connecting social interaction behavior with financial outcomes. This work reports results from one of the largest known studies connecting financial outcomes and phone-based social behavior (180,000 individuals; 2 years’ time frame; 82.2 million monthly bills, and 350 million call logs). Our methodology tackles highly imbalanced dataset, which is a pertinent problem with modelling credit risk behavior, and offers a novel hybrid method that yields improvements over, both, a traditional transaction data only approach, and an approach that uses only call data. The results pave way for better financial modelling of billions of unbanked and underbanked customers using non-traditional metrics like phone-based credit scoring.

## 1. Introduction

Humans have often been described as socio-economic beings i.e. their financial and economic behavior is intricately connected with their social behavior [1]. Not surprisingly, multiple studies have connected individual social capital with financial outcomes and credit risk [2, 3]. Since finances have a profound impact on human lives and are of vital importance to one’s livelihood, researchers have been exploring approaches to quantify financial trouble and identify methods to prevent it [4, 1].

Traditional methods of trouble prediction and credit scoring rely on historical transaction data and demographic data [5]. Credit bureaus, like Equifax and Experian, rely on financial information such as credit history, current credit use, or ratio between credit limit and outstanding balance. People having no past records are thus not able to participate in such a system. The World Bank [6] estimates that there are still two billion adults who are unbanked and lack formal financial services completely. Further, even among the ones having an account many are underbanked with banking penetration (as measured by household debt to GDP ratio) being as low as 10% for countries like India [7]. This cohort of people without credit histories also includes refugees, immigrants, students, and recent college graduates.

Most financial institutions use *capacity*, *capitals* or *collaterals* (e.g. property owned, reserve cash, debt-to-income ratio) which are static, one-time data to estimate the credit-worthiness of a customer, or use segmentation approaches which put many individuals into one unified bucket (e.g. based on age, gender, or educational qualifications) [5, 8]. Such methods are often not accurate as consumers may fail to (or choose not to) provide correct and complete demographic data, which leads to a sparse, ambiguous and unreliable dataset. Thus, there is a need for novel ways to generate credit scores and build suitable models which can iteratively learn and predict the future probability to default on credit card payments.

We posit that information about an individual’s social connections provides a natural way to augment such demographic and past behavior data for better modeling of individual financial wellbeing. Conceptually, the notion of social capital has often been connected with that of financial capital [2, 3]. Further, at an empirical basis, multiple studies have connected an individual’s position in the network, their embeddedness, and overall social behavior with financial risk [9, 2]. Given, the widespread adoption of mobile phones, even amongst the under-banked populace [10], we suggest the use of phone-based social behavioral data to augment and build better predictive models for individual credit risk. Hence, this work focuses on predicting future financial trouble by identifying socio-behavioural markers of financial trouble.

The contribution of this paper are twofold:

1. Motivate and ground the use of mobile phone based socio-behavioral data to estimate financial wellbeing.
2. To define a phone (social behavior) based Machine Learning approach to predict future propensity of financial trouble.

We do so by using a large dataset of ~180,000 individuals in Taiwan and cross validate our results over several bins in a two-year period. To the best of our knowledge, this is the first work that reports results on predicting financial trouble using phone based behavioral data for such a large scale population (~180,000 individuals) over a long time frame (2 years). Our methodology tackles highly imbalanced dataset, which is one of the most pertinent problem with modelling credit risk behavior, and identifies:

1. A call only model that works as well as a model with transaction only data with an AUCROC of ~.73
2. A novel hybrid method that improves over both a traditional transaction data only model as well as a model that uses only call data (~8% average improvement).

In the next section we survey literature on the previous work done in financial wellbeing prediction, how mobile data, when used as a proxy for social capital, can become more relevant in behavioral prediction and how it can be further expanded to questions pertaining to financial problems. We also touch upon the recent studies that use Credit Card records (CCR) data in behavioral studies.

## 2. Literature review

Financial health is critical to the wellbeing of a society and has received widespread attention from researchers and has long transcended its economics roots to be of interest to psychologists and computer scientists as well. In this section we summarize related work along four verticals. First we summarize the literature on financial trouble prediction—specifically the standard methodology followed and the evolution on these methods. Next we summarize how mobile phone data has become relevant in recent times and the myriad areas of behavioral prediction. Next, we discuss the interconnections between social capital and financial wellbeing and lastly, we present the literature on financial prediction which involves the analysis of phone and other ubiquitous sensor data.

### 2.1 Financial wellbeing as a field of study

Financial wellbeing is of utmost importance to both institutions and individuals. Institutions are now moving from crisis management to risk control. Financial outcomes for individuals can be statistically predicted from past payment history [5] using methods like time series [11], classification trees and more recently neural networks [12, 13, 14]. Yeh et al [15] used historic transaction data and compared several machine learning techniques to find Neural networks to give the best predictive power.

On a personal level, financial trouble has been linked to higher stress and is a significant factor for suicide [16] and alcohol addiction [17]. Researchers also found out that people who had better financial health had better physical health as well [14]. There is a large body of literature that connects personality traits and socioeconomic status to unreliable finical behavior with impulsiveness being correlated to spending behavior [18] and impatient people being more prone to default [19]. Financial bankruptcy has also been linked inversely to measures of social network, trust and cooperation [20].

### 2.2 Use of mobile phone in behavioral prediction

The ubiquitous nature of mobile phones in our daily lives is allowing researchers to create robust personalized models of human behaviour in social, spatial, and temporal contexts. Mobile phone usage has been used to reveal circadian rhythm patterns [21] and help identifying social signatures which are persistent over time [22]. Phone based features have been used as behavioral markers for cooperation levels [23], study individual and collective human dynamics [24, 25], infer personality [26] and understand mental health [27]. Coscia & Hausmann [28], recently showed that mobility networks can be obtained from cell-phone call networks as well. The availability of large-scale phone-based data with behavioural mapping abilities empowers researchers to not only validate and refine existing findings about health and social wellbeing but also leverage this predictive power to newer fields like understanding spending patterns and inferring financial wellbeing.

### 2.3 Social capital and its links to mobile and financial wellbeing

Social capital describes the ability of individuals or groups to access information, trust and reciprocity embedded in their social network [29]. On an individual level, social capital has been connected with higher levels of satisfaction, trust, and mental health [30]. The influence of strong and weak ties in a network has also been connected to social capital [23]. Such features have been operationalized over online social networks [31] and recently over phone networks in different contexts [32]. One’s position in a social network has been found to be associated with economic outcomes and can also improve the efficiency of economic capital [9]. For example, Van Bastelaer [3] has connected social capital with access to credit and Wang and Xiao [33] found that those with higher social support incurred less debt. On the other hand, some studies link social capital to negative externalities [34] and highlight the detrimental effects [9, 35, 36]. Thus, social capital and broadly speaking the social processes, can have significant impact on an individual’s socio-economic wellbeing [35].

### 2.4 Mobile phone and financial data

In the US alone, over 50% of smartphone users having a bank account avail mobile banking services [10]. With the availability of large amounts of detailed call and sensor data, researchers are trying to incorporate such data into financial risk prediction. Recently researchers are shifting from traditional methods involving historical transaction data to predict financial troubles to newer methods to predict trouble and credit scoring. In fact, mobile phone usage has been linked to stress and financial trouble [37] and socio-economic status has also been inferred from mobile phone activity data [38]. Researchers are now studying the interconnections between social and mobile features and spending behavior [1] and even trying to forecast financial wellbeing using mobility and call data [39, 40]. On the other hand, transaction data is also finding relevance in computational social science studies to predict consumption behavior [41] and patterns in transaction history can even identify individuals [42]. Financial bankruptcy has also been linked inversely to measures of social network, trust and cooperation [20]. Recent research has shown that credit card data like mobile phone data, can be used to detect human mobility and inform us about the preferred transitions between business categories [41] and thus create economic profiles of entire cities [43].

Building upon such trends, this work aims to analyze a large collection of longitudinal data (180,000 individuals; 2 years’ time frame; 82.2 million monthly bills, and 350 million call logs) to understand the role played by socio-behavioral features in improving the modeling of credit risk as undertaken via traditional transaction history approaches.

## 3. Dataset

This study combines several datasets for ~ 3 million customers of a major bank and combines it with mobile data for a subset of same individuals. A summary of the data considered is shown in Table 1.

**Table 1:** Dataset summary for various data sources used in the study.

Dataset	Description	# records
**Bill Data**	Records of bill generated for each month	3.6 million accounts, 82.2 million monthly bills
**Transaction Data**	Day to day transaction for each individual including purchase type and location of purchase	2.3 million accounts, 190 million transactions
**Demographic Data**	The account holder which is recorded and regularly updated by the bank.	1.6 million
**Mobile Data**	Call related data including hashed remote number called and doesn’t include any private information	180, 000 users, 350 million call logs

The datasets are explained further in the following subsections.

### 3.1 Bill data

The bill data contain about 82.2 million monthly bills belonging to 3.6 million credit accounts from a major bank in Taiwan. For each account, the basic bill records, such as bill amounts, maximum-allowed credit amounts, and the paid amounts were collected for each month from January 2014 to December 2015. Customer names were removed and only anonymized identifiers were used for analysis. Besides all the basic bill records, the bank also marked the Pay Rating for each customer in each month based on his or her paying behavior in the previous month with the following definition (Table 2):

**Table 2:** Bill data consumer rating summary.

Rating	Description	% of Population
**0**	No consumption	52.35%
**1**	Pay full amount on time	39.85%
**2**	Pay full amount—not on time	0.76%
**3**	Pay minimum	5.98%
**4**	Pay minimum amount late	0.55%
**5**	Pay less than minimum amount	0.06%
**6**	Not pay	0.46%

In order to reduce the number of meaningful dependent variable we decided to make it binary. Based on this Pay Rating records, a customer is considered having financial trouble in a specific month if she fails to pay even the minimum amount to avoid a late fee or not at all i.e. got a Pay Rating 4, 5, or 6. This new “trouble” variable will be used as the outcome variable in our prediction model (Table 3). We also tried including Pay Rating 2 (paying full amount—not on time) into the definition of “trouble”, which leads to worse predicting performance as will be shown in Appendix A in S1 File. It might suggest that people have Pay Rating 2 are just missing their deadlines by accident, rather than having financial troubles, and hence are harder to be predicted in this application. However, although the results are worse, the trend is still the same, i.e., call features still improve the performance and the combined model outperforms homogeneous models, as will be discussed in following sections.

**Table 3:** Trouble variable summary.

Trouble	Description	% of Population
**0**	Avoids late payment	98.94%
**1**	Makes Payment late or no Payment	1.07%

### 3.2. Transaction data

**Fig 1:**
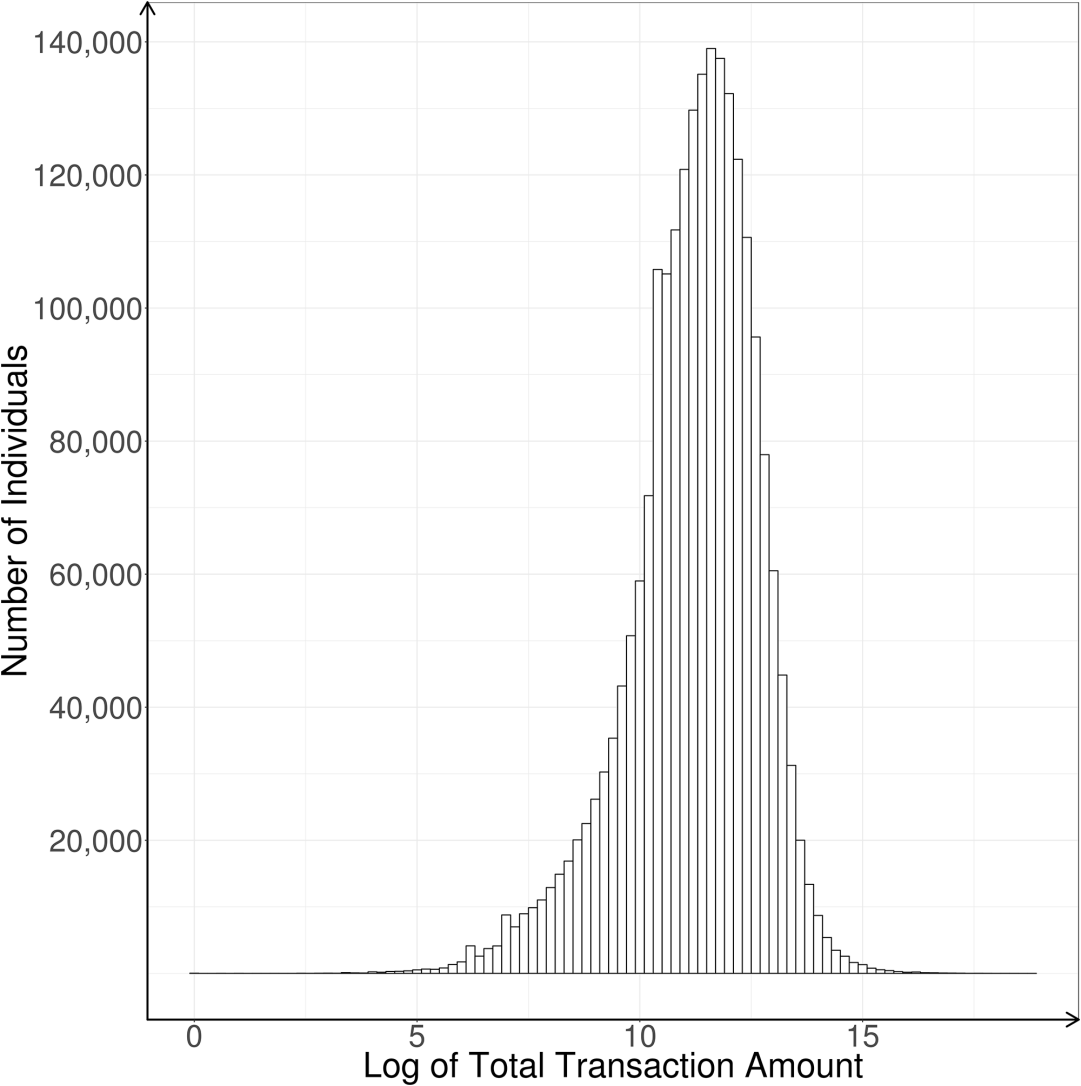
Distribution of credit card transaction amounts (on a log scale).

The transaction data contain about 190 million transactions made by 2.3 million credit accounts within the same 2-year time interval as specified in the bill data. The transactions follow a standard log normal distribution (Fig 1). The same anonymized identifications are used to map customers between the bill and the transaction datasets. The transaction data include the following attributes:

- Transaction date (in year-month-day format)
- Transaction amounts
- Merchant shop names
- Unique merchant code given by the bank
- Merchant country and city
- Merchant category codes (4-digit MCC code which explains the category of the merchant e.g. one for hotels, one for office supply stores, etc.)

A summary of statistics of the transaction data can be found in Appendix B in S1 File. The attributes listed above are further processed into other calculated measures or indices to be used as features in our prediction model, as will be described in Section 4.

### 3.3. Demographic data

The basic demographic features and the account properties of about 1.6 million customers are also provided by the bank. The same anonymized identifications are again used to map customers between different datasets. The demographic data include the following attributes:

- Education level
- Gender
- Annual income level
- Marital status
- Position in occupation
- Post code of address

A summary of statistics of the demographic data can be found in Appendix B in S1 File.

### 3.4. Call data

We also have access to 350 million call logs of about 180 thousand customers within 22 months from January 2014 to October 2015. The customer mapping are made by the bank via the associated anonymized identifications. These call data contain:

- Timestamp of beginning, off-hook, and idle time of each call (in Unix time)
- Duration of each call
- Remote number of each call
- Whether or not the remote number was saved in the contact list of the phone

The content of these calls were not recorded and only the call metadata (time, duration, anonymized person ids) were used to create the metrics. A summary of statistics of the call data can be found in Appendix B in S1 File. From these per-call logs we constructed call-related features for each customer including volumes of calls or proportion of calls with some specific properties, as will be described below.

### 3.5. Data preprocessing and cleaning

In order to ensure integrity and completeness of the data, we removed 35 (< 0.01%) accounts which have more than one bills in at least one month, and then removed 14,027 (0.39%) accounts which have blank Pay Ratings. After removal, there were ~82 million bill records belonging to 3.6 million credit accounts. From the transaction data, we removed 139,314 (5.97%) accounts which map to more than one customers and the remaining data contained about 164 million transactions made by 2.2 million credit accounts each map to a single customer. The bill data and transaction data are then merged together and the resulting joint data consisted of about 2.2 million customers.

From the call data of each customer, we removed call records with invalid timestamps (e.g., records without idle time or records with off-hook time occurring after idle time), abnormal remote numbers (e.g., records without remote number or records with remote number shorter than 3 digits), or abnormal durations (e.g., records with duration longer than 6 hours). The resulting call data contain about 350 million call records belonging to 180 thousand customers.

We then merged all our datasets to finally get ~180,000 records of transaction history as well as call records.

## 4. Feature identification—Definitions and rationale

We use sliding-window mechanism to define our predicting periods. Concretely, we use features in the previous 9 months to predict whether a customer will have financial trouble in the current month, making each window to be 10-month long. In this problem setting, we can use both period-specific features and consistent features as described below.

### 4.1. Period-specific features

For each possible predicting period, we use the transaction and call data collected in the first 9 months of the period to construct features as follows.

#### 4.1.1. Transaction features

Based on a review of features defined in related literature on quantifying user behavior using financial transactions [39], we construct the following statistical features for each customer (Table 4):

**Table 4:** Transaction based features.

Metric	Formula	Description
**Min/Max/Total**	Min /Max / Log (T)	Quantifies the overall transaction activity. Note: T can be number of transactions /transaction amount
**Coefficient of Variation**	$\frac{\text{standard}\;\text{deviation}\;\text{of}\;\text{T}}{\text{Average}\;\text{T}}$	Quantifies the spread of T.
**Weekly Ratio**	$\frac{\text{T}\;\text{during}\;\text{weekend}}{\text{Total}\;\text{T}}$	Quantifies the preference to spend on weekdays.
**Holiday Ratio**	$\frac{\text{T}\;\text{during}\;\text{holiday}}{\text{Total}\;\text{T}}$	Quantifies the preference to spend on holidays.
**Domestic Ratio**	$\frac{\text{T}\;\text{in}\;\text{Taiwan}}{\text{Total}\;\text{T}}$	Quantifies the preference to spend in Taiwan vs foreign.
**MCC Ratio**	$\frac{\text{T}\;\text{in}\;\text{a}\;\text{given}\;\text{MCC}}{\text{Total}\;\text{T}}$	Quantifies the likelihood to spend on a given category as described below.
**Salary Days Ratio**	$\frac{\text{T}\;\text{in}\;\text{first},\text{middle},\;\text{or}\;\text{last}\;10\;\text{days}}{\text{Total}\;\text{T}}$	Quantifies the likelihood to spend within 10 days of salary credit (i.e. first 10 days of the month)
**Diversity**	∑_*j*_ *p*_*j*_ log_*b*_ *p*_*j*_ *p*_*j*_ = percentage of T in MCC ‘*j*’, *b* = total number of MCC	Quantifies the evenness of spending across MCC* category bins
**Loyalty**	$\frac{\text{T}\;\text{top}\;3\;\text{categories}}{\text{Total}\;\text{T}}\;\times \;100$	Quantifies the preference of spending in top 3 MCC* category bins

*where T can be number of transactions /transaction amount MCC categories [44]:
Business Services Utilities Service providers Retail Stores (Grocery stores, Supermarkets Government services

#### 4.1.2 Call features

At a conceptual level, social capital has been connected with an individual’s relative position in the network [34] On a more granular level, the influence of strong and weak ties in a network has been connected to social capital [34, 9]. Similarly, prior research links the frequency of interactions with an individual’s network with their social capital [34, 45]. Further, social capital has been connected to reciprocity of contacts and the ease of availability [46, 47].

Such features have been operationalized over online social networks [31] and recently over phone networks in different contexts [26]. Hence, based on a survey of existing literature on quantifying user behavior using phone transactions (e.g. [1, 23, 26, 27]), we construct the following statistical features for each customer in Table 5.

**Table 5:** Call/Call duration data based features.

Metric	Formula	Description
**Social Activity**	Log (Comm*)	Quantifies the overall communication activity.
**Diurnal Activity Ratio**	$\frac{\text{Comm}*\;\text{during}\;\text{phase}\;1}{\text{Total}\;\text{Comm}*}$	Quantifies the circadian rhythm the ratio of communication taking place in four six hour phases.
**Weekly Activity Ratio**	$\frac{\text{Comm}*\;\text{during}\;\text{weekend}}{\text{Comm}*\;\text{during}\;\text{weekday}}$	Quantifies the difference between “work” week and the more social weekend behavior.
**Strong/Weak Ties Engagement Ratio**	$\frac{\text{Comm}*\;\text{top}/\text{bottom}\;\text{third}\;\text{contacts}}{\text{Total}\;\text{Comm}}\times 100$	Quantifies the communication effort that a user devotes to her top/bottom third contacts. We do this for both known, unknown and overall contacts.
**Daily Inter-event Time**	$\frac{\text{Time}\;\text{iterval}\;\text{between}\;\text{two}\;\text{comm}*\;\left(\text{hours}\right)}{\#\;\text{of}\;\text{comm}*\;\text{in}\;\text{the}\;\text{day}}$	Quantifies the frequency of comm* in a day.
**Diversity**	∑_*j*_ *p*_*ij*_ log_*b*_ *p*_*ij*_ *p*_*ij*_ = percentage of engagements made by individual ‘*i*’ to contact ‘*j*’, *b* = total number of comm*	Quantifies the evenness of engagements across contacts
**Contact Engagement Ratio**	$\frac{\text{Comm}*\;\text{with}\;\text{saved}\;\text{contacts}}{\text{Total}\;\text{Comm}*}$	Quantifies the reception of comm* from contacts i.e. numbers in the phonebook of the individual (can also be landline numbers)
Comm* **Latency**	$\frac{\text{Ringing}\;\text{during}\;\text{phase}\;1}{\text{Total}\;\text{comm}*}$	Quantifies the latency in comm* (calculated only for incoming and missed)
Comm*1-Comm*2 Ratio	$\frac{\text{Incoming}\;\text{comm}*1}{\text{Outgoing}\;\text{comm}*2}$	Quantifies the likelihood of replying to the communication during a given time period.

*where comm can be incoming/outgoing/missed/total calls

### 4.3 Demographic features

As commonly used in credit scoring systems, we also collected the following demographic features (Table 6):

**Table 6:** Demographic based features.

Variable	Description
**Gender**	1: male 2: female
**Education Level**	1: doctoral; 2: master; 3: bachelor; 4: college; 5: high school; 6: others
**Annual income level**	(1: below 1 million; 2: 1 ~ 3 million; 3: 3 ~ 5 million; 4: above 5 million)
**Marital status**	(1: married; 2: not married; 3: divorced)
**Position in occupation**	(1: responsible persons; 2: executives; 3: mid-level executives; 4: normal employees; 5: others)
**Number credit cards**	Number of open credit cards across all banks

## 5. Results

### 5.1. Methodology

We considered a binary classification problem in which the outcome is defined as whether or not a customer will have financial trouble in each month using three different sets of features: using only call, only transaction and the third combining both. The model trained in a specific window will be tested in the next window that is one-month shifted from the training window. For example, we use features collected from January 2014 to September 2014 and the outcome in October 2014 to build a prediction model, and evaluate the performance of the model using the features collected from February 2014 to October 2014 and the outcome in November 2014.

The outcome considered in this work leads to extremely imbalanced datasets in which less than 3% of customers are considered having financial trouble in any given bill month. To mitigate the effects of accuracy paradox due to such imbalance, the majority class (i.e. customers considered not having financial trouble) is randomly sampled to produce a balanced training data, and the obtained model is then tested using realistic imbalanced settings in the testing window. All possible testing windows are denoted as below:

- P01: 2014/02-2014/10
- P02: 2014/03-2014/11
- P03: 2014/04-2014/12
- P04: 2014/05-2015/01
- P05: 2014/06-2015/02
- P06: 2014/07-2015/03
- P07: 2014/08-2015/04
- P08: 2014/09-2015/05
- P09: 2014/10-2015/06
- P10: 2014/11-2015/07
- P11: 2014/12-2015/08
- P12: 2015/01-2015/09
- P13: 2015/02-2015/10
- P14: 2015/03-2015/11

All models are built using eXtreme Gradient Boosted Models (XGBoost). Xgboost is a boosting ensemble method which sequentially trains models with each subsequent model seeking to minimize residuals weighted by the previous model’s errors using a given loss function [48]. The balancing process for each training window are repeated 10 times to get the average feature importance. All models are applied on the testing window to get the average testing performance. The performance is measured by area under the receiver operating characteristic curve (AUCROC), and the feature importance are estimated in terms of (normalized) relative influence. We use the R-based implementation of Xgboost for all our tests [49]. We considered the fact that AUCROC can be a useful metric in classification scenarios when a trade-off between true positive rate and false positive rate is of vital interest. (Note: The baseline for ROC was taken to be 0.500 irrespective of the cross-validation.).

### 5.2. Testing results

We built models for each of the fourteen training periods, applied them to the corresponding testing windows. The averaged results are (Table 7):

**Table 7:** Testing results—Predicting financial trouble as a function of different feature sets.

	Call +Transaction + Demographics	Call	Transaction + Demographics
AUCROC	0.781	0.725	0.731
Recall	0.731	0.679	0.689
Accuracy	0.687	0.649	0.652

**Fig 2:**
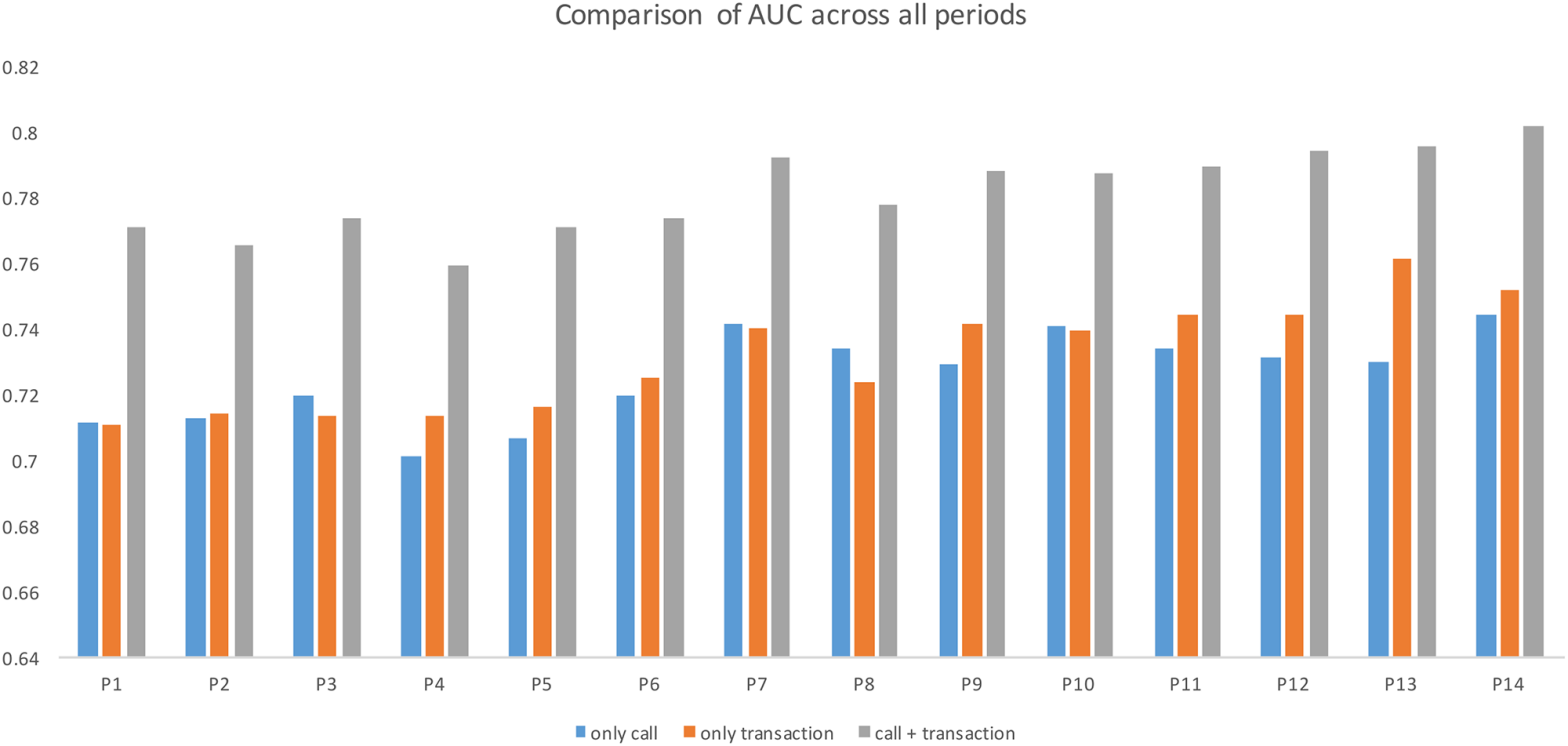
AUCROC comparison across all periods.

It can be seen that, in all cases, adding call features can improve the predicting power of the model. We also note that the results are consistent over each period. Results of all 14 predicting periods are showed in Fig 2.

We also perform a pairwise t-test to check whether the improvements in AUCROC are significant when we take the combined model. We get the following results (Table 8):

**Table 8:** T-test for AUCROC.

Testing call + transaction model against:	T score (P value)
Only transaction model	28.422 (4.342e-13)
Only call model	33.248 (5.802e-14)

In both cases we reject the null hypotheses (P<0.001) and find that the combined model is an improvement over the homogeneous models.

From Table 9, an interesting thing to note is that the T-test fails (p-value > 0.5) when we test the transaction only vs the call only model in terms of both AUCROC and the accuracy scores. This indicates that a call only model can perform almost as well as a transaction only model which contains no transaction records. This result suggests that a call only model can replace a model made of transaction history and produce equivalent (if not better) results.

**Table 9:** T-test comparing call only and transaction only model.

Testing call vs transaction model	T score (P value)
AUCROC	-2.1056 (0.05525)
Accuracy	-1.027 (0.3231)

### 5.3. Feature importance

In the setting of including call features, the overall importance of call features accounts for about 60% among all features in all different predicting periods. We rank all features based on their average importance in all 10 iterations in each predicting period, and then take the average rank over all 14 periods. To gain further insight into the features identified and their relative effect on the propensity of financial trouble we undertook post-hoc correlation analysis between the trouble variable and the different features identified. The following Table 10 shows the top-10 features for each category, as well as the sign (positive or negative) of their Pearson’s correlation with the outcome variable (having financial trouble or not). Note that the correlations were significant (p value < 0.05) for all the listed features except COV (Coefficient of Variation). See Table 10.

**Table 10:** Top-10 features for each category, as well as the sign (positive or negative) of their Pearson’s correlation with the outcome variable (having financial trouble or not).

Rank	Call +Transaction + Demographics	Call	Transaction + Demographics
1	Inter-event time (incoming)	Inter-event time (incoming)	# months with at least one transaction
2	# months with at least one transaction	Incoming Latency (daytime)	Coefficient of variation
3	MCC ratio (Business Services)	Missed call Latency	Weekend ratio (# transactions)
4	Coefficient of variation	Contact engagement ratio (Outgoing)	Mean transaction across all banks
5	MCC ratio (Retail Stores)	Interevent time (Outgoing)	MCC ratio (Retail Stores)
6	Mean transaction across all banks	Incoming Latency (morning)	MCC ratio (Business Services)
7	Missed Call Latency (total)	Landline engagement ratio (outgoing)	Domestic Ratio (# transactions)
8	# of opened credit cards	Contact engagement ratio (total)	MCC ratio (Utilities)
9	Domestic Ratio (# transactions)	Contact engagement ratio (incoming)	Maximum transaction amount
10	Incoming Latency (daytime)	Incoming Latency (night)	# of opened credit cards

Red indicates negative impact while green indicates positive taken over all the periods. COV is white as it exhibited positive correlation in some periods while being negative in others.

#### 5.3.1 Interpreting call based features

Each of the associations identified above is correlational rather than causation-driven. Hence, we are not able to identify the direction of the effect. Further, there remains multiple ways to interpret the features. Hence the associations noted are meant to help interpret the predictive models identified in the preceding sections rather than being prescriptive in their own right. In future work, we would like to design intervention studies and/or conduct follow up interviews to understand the nuances of each association. With these caveats in place, we discuss here the general trends observed in the associations.

As we can see from Table 10 the most significant feature for both the calls only model and the hybrid model is Inter-event time (incoming) while Inter-event time (outgoing) also features at rank 5 in the top ten. Inter-event time was the average time between two communications (here incoming calls) in a day. We see that it is negatively correlated to the response variable indicating that as the time increases between two calls the propensity to default decreases i.e. people who make or get more frequent calls are more likely to be in financial trouble. This is an interesting result and may be associated with the darker side of social capital. Adler and Kwon [35] argue that in-group members may sometime over-embed the actor and block access to new information. Again, social capital presents risks of negative externality as outlined by Coleman [34]. It may so happen that the in-group of the troubled individual may itself be in financial trouble and exploit the other and such a situation may lead to tragedy of commons for the aggregate. However, the balance of positive and negative externalities are dependent on the beliefs and source of the social capital so we leave these questions open to further investigation but at the same time corroborate prior literature that suggests that social capital can sometimes be detrimental [9, 35, 36].

Another interesting feature is the latency in picking up calls whether it be incoming during daytime (rank 2) or morning (rank 6), missed (rank 3) during daytime or incoming latency at night (rank 10). Latency was defined as the ringing time before the call is picked up or gets dropped by the total no of calls (incoming/missed). This implies that people who have trouble might take more time to pick up calls. While multiple explanations are possible, this could in part be attributed to the reluctance to engage with others (as above) or even fearing calls from certain contacts and/or banking agencies.

A third important feature is the contact engagement ratio (rank 4 and 8.) It is defined as the ratio of total communication spent with contacts saved in the person’s phonebook. This is negatively correlated to the response variable indicating that people with no trouble tend to talk to known people more and might not engage with unknown numbers. Another way to interpret this is that preferentially connecting with stronger ties (higher bonding social capital [50]) is associated with lesser financial trouble.

#### 5.3.2 Interpreting transaction based features

The most significant transaction based feature is number of months with at least one transaction. This is interesting as people who have more number of months with transaction seem to be less in trouble indicating that people who use their credit cards regularly are actually more conscious of the use and thus fell obligated to pay on time. It may so happen that people who rarely use their cards, end up missing the deadline.

The second and third most significant feature is MCC ratio (business services) and MCC ratio (retail services). These features indicate the ratio of transactions made at a particular type of stores and gives us insight into the difference in spending behavior of troubled individuals. People who spend more on business services are more likely to be in trouble while people who spend more on utilities might have less trouble. This may be due to the fact that business services bills are often larger than the essential groceries bill and people might have a hard time paying back the non-essential or larger expenses. Also business utilities are over and above the basic necessities and may include unnecessary expenses.

Finally, the third most important feature is the domestic transaction ratio i.e. the amount spent in Taiwan compared to all transaction. People who spend mostly in Taiwan tend to have less trouble indicating that people often overuse their cards while travelling abroad. This could simply due to higher expenses incurred with foreign travel but could also be associated with a lack of awareness regarding the exchange rate or the exchange fee levied on such transactions.

#### 5.3.3 Interpreting demographic based features

The most important demographic based feature is the number of credit cards being opened. This is again negatively correlated to the trouble again indicating that people are conscious of the credit cards they keep and pay bills timely.

We notice that several features are common in the hybrid model and the respective homogenous models. The transaction only model shares 7 common features with the hybrid model while call only model shares 2. Thus, similar set of features are present in the hybrid and the homogenous models indicating that we can use various combinations of the features when subject to availability of robust data or under computational constraints.

Given that we carry out these interpretations post hoc, we focus on triangulating and identifying general trends across the two analysis methods (correlation and classification) rather than establishing hard associations between specific variables. These interpretations are only to aid and steer the discussion on the possible implications of such an association between mobile data and social behaviour. The main objective of the paper is to motivate the use of mobile phone based socio-behavioral data to estimate financial wellbeing.

## 6. Discussion

Overall, the results suggest that phone-based socio-mobile features can have significant predictive power over an individual’s credit risk. This can have important implications for individuals as well as organizations. At the same time, they highlight privacy and ethical considerations as well as opportunities for future work.

All data used in this study were hashed and anonymized and at no point actual phone numbers or call contents were available to the personnel undertaking the analysis. All the transaction data and bill data were provided by the bank and was again hashed. These behavior-to-outcome connections also have implications for the privacy of users [39]. We hope that the results presented here will raise user awareness on the implications of sharing phone data with a wide variety of stakeholders and mobile apps. The findings of a public study like this one are critical to motivating a discussion on the right policy parameters surrounding phone and by extension social data as there are no standard guidelines about the use of mobile-phone data.

The obtained results highlight the importance of social features in predicting the financial outcomes of individuals. The given models are applicable to both people with no transaction history (the call only model) and people with limited transaction history (hybrid model). This work leverages passively collected data from mobile phones, something which most of the world now has access to. We would also like to point out that most of the call features can be created using data from a feature phone as well and is again useful in a demographic where smartphone might be a luxury. Most financial institutions use static, one-time data to estimate the credit-worthiness of a customer, or use segmentation approaches which put many individuals into one unified bucket. The emergence of individual transaction profiles for each customer now allows for creation of rich personalized models of each user’s behavior that can be used to predict their behavior. Also we note that the kind of analysis described here can be done incrementally during the month before the payment deadlines, thus allowing preemptive remedies *before* a user starts missing her payments and becomes delinquent.

We also highlight some insights into the nature of social capital and how it might be both detrimental and beneficial to coping with financial trouble. While some of the features (e.g. inter-event time for calls) were found to be bad for an individual’s financial wellbeing, others such as the latency in picking up calls was found to be positively associated with financial wellbeing. While each of these results needs to be evaluated in more detail in future work, it motivates the use of large-scale “in-the-wild” social/phone based behavioral markers to study financial wellbeing. In that sense, it also adds to the existing literature surrounding the use of smartphones in assessing social capital.

Considering that many major banking apps (e.g. Bank of America, Citi bank) already require permissions to access call data, it is plausible for them to integrate call-based data to refine their prediction models. In some large economies like India it is now mandatory to link all bank accounts and phone numbers to a central unique identification number (called AADHAR), suggesting that in future phone and financial data could be integrated to create hybrid models. The availability of data in this case would clearly require policy regulations. Lastly banking in many developing countries are based on microfinancing institutions which largely carry out transactions over the mobile phone giving the firm access to both banking and call data. Thus, the advent of mobile banking and centralized data collections can make availably of large and robust data sets easily available and make hybrid models such as the one studied here quite feasible.

Finally, with the appropriate checks and balances in place, the observations presented here could be used in the future to provide feedback and nudges to the individuals themselves. For example, a sudden decrease in social activities, or change in rhythms of social behavior, could be used to create customized alerts asking the individual to be extra careful with their financial payments for the month. Of course the final decision about behavior change must always remain with the user: they may choose to ignore the message or use it as a reminder to moderate their behavior.

## 7. Conclusion and future work

This paper proposes alternative methods to traditional credit scoring and provides a novel way to predict the future propensity of an individual to default on her credit card bill using 9 months of historic data with an AUCROC of ~.78. This is fundamentally different from the standard approaches popular with credit bureaus and performs better than comparable transaction-based approaches. It goes on to show that call data can be an important signal of a person’s financial troubles and reinforces them as a proxy for socio-economic behavior.

As the world is moving towards smartphones, wearable and more immersive and ubiquitous technology we would consider incorporating data streams collected to further ascertain the impact of socio-behavioral features on financial wellbeing. Such interconnections could yield insights into fundamental human behavior while also yielding more accurate risk assessment. Lastly, we would also like to extend and adapt this study to developing economies where a study like this can make a true impact.

## Supporting information

S1 File Contains analysis after including Pay Rating 2 in the trouble definition and summary statistics of selected attributes of transaction, demographic, and call datasets. (DOCX)

S1 Fig AUCROC comparison across all periods if including Pay Rating 2 as trouble. (TIFF)
