# Supplementary material for: Predicting financial trouble using call data—On social capital, phone logs, and financial trouble
Source: PLoS One. 2018 Feb 23;13(2):e0191863. doi: 10.1371/journal.pone.0191863 (PMC5825009; doi:10.1371/journal.pone.0191863)
Supplement: S1 File — (DOCX) [file pone.0191863.s001.docx]

# S1 Appendix A

By including Pay Rating 2 (Pay full amount – not on time) into the definition of “trouble”, we get a slightly higher proportion of positive outcome variables (Table A1). Note that although the proportion of Pay Rating 2 is small (0.76%), it is relatively high compared to Pay Rating 4, 5, or 6 (at most 0.55%).

Table A1: Trouble variable summary if considering Pay Rating 2 as trouble

| **Trouble** | **Description** | **% of Population** |
| --- | --- | --- |
| **0** | Avoids late payment | 98.18% |
| **1** | Makes Payment late or no Payment | 1.83% |

Using this new outcome variable and follow the same procedures as described in Section 6, we get the averaged testing results (Table A2):

Table A2: Testing results if including Pay Rating 2 as trouble

|  | **Call +Transaction + Demographics** | **Call** | **Transaction + Demographics** |
| --- | --- | --- | --- |
| **AUCROC** | **0.716** | 0.679 | 0.662 |
| **Recall** | **0.674** | 0.648 | 0.621 |
| **Accuracy** | **0.642** | 0.612 | 0.615 |

It can be seen that, there is about a 5% degradation consistently in all cases. The relatively worse results might suggest that, people have Pay Rating 2 are just missing their deadlines by accident, rather than having financial troubles, and hence are harder to be predicted in this application. Note that, although the results are worse, the trend are still the same: the combined model outperforms both homogeneous models. The results are also consistent over each period. Results of all 14 predicting periods are showed in Fig A1.

Fig A1 AUCROC comparison across all periods if including Pay Rating 2 as trouble

While the results are worse, the feature ranking are similar as before (Table A2):

Table A2: Top-10 features for each category, as well as the sign (positive or negative) of their Pearson’s correlation with the outcome variable (having financial trouble or not). *Orange indicates negative impact while grey indicates positive taken over all the periods*.* COV is white as it exhibited positive correlation in some periods while being negative in others.

| **Rank** | **Call +Transaction + Demographics** | **Call** | **Transaction + Demographics** |
| --- | --- | --- | --- |
| 1 | Inter-event time (incoming) | Inter-event time (incoming) | # months with at least one transaction |
| 2 | MCC ratio (Retail Stores) | Contact engagement ratio (outgoing) | MCC ratio (Retail Stores) |
| 3 | # months with at least one transaction | Missed call Latency | Weekend ratio (# transactions) |
| 4 | MCC ratio (Business Services) | Incoming Latency (daytime) | Coefficient of variation |
| 5 | Domestic Ratio (# transactions) | Contact engagement ratio (total) | Mean transaction across all banks |
| 6 | Coefficient of variation | Landline engagement ratio (outgoing) | MCC ratio (Business Services) |
| 7 | Contact engagement ratio (outgoing) | Contact engagement ratio (incoming) | Domestic Ratio (# transactions) |
| 8 | Missed call Latency | Interevent time (outgoing) | MCC ratio (Utilities) |
| 9 | Incoming Latency (daytime) | Missed call ratio (daytime) | # of opened credit cards |
| 10 | Mean transaction across all banks | Incoming Latency (morning) | MCC ratio (Department Stores) |

# S1 Appendix B

Summaries of statistics of selected attributes of transaction, demographic, and call datasets are presented in the following tables.

Table B1: A summary of various transaction attributes and their descriptive statistics

| **Transaction Amount** | **Number of Transactions Under Different Condition** |
| --- | --- |
| Min.: 0 1st Qu: 445 | Weekends: 50,062,809 (31.08%) |
| Median: 750 | Holidays: 5,647,389 (3.51%) |
| Mean: 2371 | Domestic (Taiwan): 151,403,281 (94.01%) |
| 3rd Qu: 1569 |  |
| Max.: 82,963,905 |  |

Table B2: A summary of various per person statistics of transaction data

| **Total Transaction Amount (Per Person)** | **Total Number of Transaction (Per Person)** | **Number of Month with at Least One Transaction (Per Person)** |
| --- | --- | --- |
| Min.: 0 | Min.: 1 | Min.: 1 |
| 1st Qu: 34,986 | 1st Qu: 16 | 1st Qu: 8 |
| Median: 89,203 | Median: 43 | Median: 16 |
| Mean: 174,225 | Mean: 73.48 | Mean: 14.94 |
| 3rd Qu: 201,182 | 3rd Qu: 98 | 3rd Qu: 23 |
| Max.: 151,343,115 | Max.: 10,826 | Max.: 24 |

Table B3: A summary of various demographic attributes and their descriptive statistics

| **Education Level** | **Gender** | **Annual Income Level** |
| --- | --- | --- |
| Doctoral: 17,876 (1.09%) | Female: 879,146 (53.59%) | Below 1 million: 1,436,490 (87.57%) |
| Master: 153,059 (9.33%) | Male: 761,233 (46.40%) | 1 ~ 3 million: 170,937 (10.42%) |
| Bachelor: 545,003 (33.22%) |  | 3 ~ 5 million: 15,768 (0.96%) |
| College: 367,129 (22.38%) |  | Above 5 million: 13,446 (0.82%) |
| High School: 378,256 (23.06%) |  |  |
| Others: 170,793 (10.41%) |  |  |
| **Marital Status** | **Position in occupation** | **Number credit cards** |
| Married: 973,889 (59.37%) | Executives: 203,695 (12.42%) | Min.: 0 |
| Not married: 658,190 (40.12%) | Normal employees: 842,475 (51.36%) | 1st Qu:2 |
| Divorced: 561 (0.03%) | Others: 584,710 (35.64%) | Median: 3 |
|  |  | Mean: 4.19 |
|  |  | 3rd Qu: 5 |
|  |  | Max.: 463 |

Table B4: A summary of various per person statistics of call data

| **Total Duration of Calls (Minutes** **Per Person)** | **Average** **Duration of Calls (****Minutes Per Person)** | **Average Rin****gtone Duration (Seconds Per Person)** |
| --- | --- | --- |
| Min.: 0.02 | Min.: 0.02 | Min.: 0.00 |
| 1st Qu: 218.40 | 1st Qu: 1.09 | 1st Qu: 11.80 |
| Median: 940.17 | Median: 1.44 | Median: 14.06 |
| Mean: 2191.82 | Mean: 1.75 | Mean: 14.65 |
| 3rd Qu: 2682.43 | 3rd Qu: 1.98 | 3rd Qu: 16.80 |
| Max.: 166767.67 | Max.: 56.33 | Max.: 198.36 |
| **Total Number of Calls (Per Person)** | **Total Number of Contacts** **(P****er Person)** |  |
| Min.: 1 | Min.: 1 |  |
| 1st Qu: 218 | 1st Qu: 54 |  |
| Median: 864 | Median: 147 |  |
| Mean: 1516 | Mean: 199.5 |  |
| 3rd Qu: 2048 | 3rd Qu: 274 |  |
| Max.: 37243 | Max.: 8897 |  |
