# Supplementary figures and images for: Predicting financial trouble using call data—On social capital, phone logs, and financial trouble
Source: PLoS One. 2018 Feb 23;13(2):e0191863. doi: 10.1371/journal.pone.0191863 (PMC5825009; doi:10.1371/journal.pone.0191863)

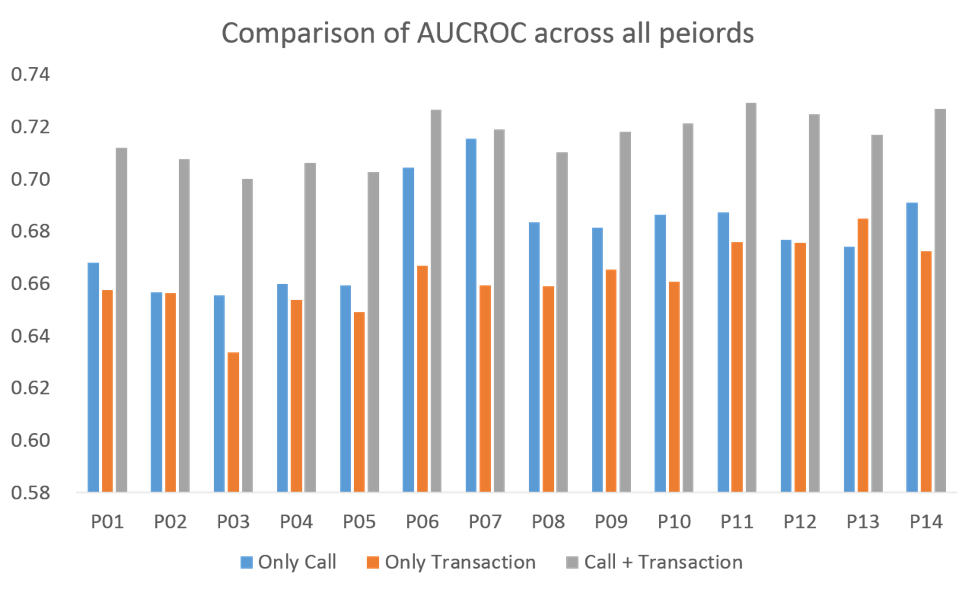

Supplement: S1 Fig — (TIFF) [file pone.0191863.s002.tiff]
